# *Legionella* RavZ Plays a Role in Preventing Ubiquitin Recruitment to Bacteria-Containing Vacuoles

**DOI:** 10.3389/fcimb.2017.00384

**Published:** 2017-08-28

**Authors:** Tomoko Kubori, Xuan T. Bui, Andree Hubber, Hiroki Nagai

**Affiliations:** ^1^Department of Infectious Disease Control, Research Institute for Microbial Diseases, Osaka University Suita, Japan; ^2^Department of Microbiology, Graduate School of Medicine, Gifu University Gifu, Japan

**Keywords:** *Legionella*, *Salmonella*, ubiquitin, vacuole, effector proteins, autophagy

## Abstract

Bacterial pathogens like *Salmonella* and *Legionella* establish intracellular niches in host cells known as bacteria-containing vacuoles. In these vacuoles, bacteria can survive and replicate. Ubiquitin-dependent selective autophagy is a host defense mechanism to counteract infection by invading pathogens. The *Legionella* effector protein RavZ interferes with autophagy by irreversibly deconjugating LC3, an autophagy-related ubiquitin-like protein, from a phosphoglycolipid phosphatidylethanolamine. Using a co-infection system with *Salmonella*, we show here that *Legionella* RavZ interferes with ubiquitin recruitment to the *Salmonella*-containing vacuoles. The inhibitory activity is dependent on the same catalytic residue of RavZ that is involved in LC3 deconjugation. In semi-permeabilized cells infected with *Salmonella*, external addition of purified RavZ protein, but not of its catalytic mutant, induced removal of ubiquitin associated with *Salmonella*-containing vacuoles. The RavZ-mediated restriction of ubiquitin recruitment to *Salmonella*-containing vacuoles took place in the absence of the host system required for LC3 conjugation. These observations suggest the possibility that the targets of RavZ deconjugation activity include not only LC3, but also ubiquitin.

## Introduction

*Legionella pneumophila* is a Gram-negative bacterial pathogen that has a wide variety of eukaryotic hosts, ranging from amoebas to humans (Isberg et al., 2009). Host phagocytosis normally mediates entry of *L. pneumophila* into cells. Immediately after entry, *L. pneumophila* remodels the phagosome into a replicative niche, often referred to as a *Legionella*-containing vacuole (LCV). In order to establish the niche, *L. pneumophila* translocates ~300 effector proteins from bacteria into host cells via the specialized Dot/Icm type IV secretion system (T4SS), and by the coordinated function of these effector proteins it modulates a variety of host cellular processes (Ensminger and Isberg, 2009; Isberg et al., 2009; Hubber and Roy, 2010).

Similarly to other bacterial pathogens, *L. pneumophila* utilizes or modulates host ubiquitin pathways for its own benefit (Hubber et al., 2013; Zhou and Zhu, 2015). It was reported that *L. pneumophila* recruits ubiquitin to LCVs by a process depending on the Dot/Icm T4SS and bacterial protein synthesis (Dorer et al., 2006). The ubiquitin associated with LCVs is targeted for host proteasomal degradation, which was suggested to generate amino acids required for bacterial intracellular growth (Price et al., 2011). *L. pneumophila* possesses a large array of effector proteins involved in the modulation of the host ubiquitin system. Currently, several effector proteins have been found to possess the F-box and U-box domains implicated in E3 ubiquitin ligase activity (Kubori et al., 2008; Hubber et al., 2013). A recent structural analysis revealed that the bacterial protein SidC represents a novel family of E3 ubiquitin ligases (Hsu et al., 2014) containing a unique PI(4)P-binding domain (Luo et al., 2015). SidC associates with LCVs through its high affinity PI(4)P-binding domain (Weber et al., 2006; Ragaz et al., 2008; Dolinsky et al., 2014) and has a direct or indirect role in ubiquitin recruitment to LCVs in early stages of infection (Horenkamp et al., 2014). *L. pneumophila* SidE family proteins exhibit not only deubiquitinase activity (Sheedlo et al., 2015) but also unusual ubiquitin ligase activity independent of the canonical E1 and E2 enzymes (Qiu et al., 2016; Kotewicz et al., 2017).

Autophagy is a conserved eukaryotic catabolic process for breakdown of intracellular components such as proteins or organelles. The target components are sequestered into a membrane-bound compartment called the autophagosome, which eventually fuses with a lysosome, a digestive compartment (Kuballa et al., 2012). Ubiquitin-like conjugation systems including Atg (Autophagy-related genes) proteins are crucially involved in autophagy (He and Klionsky, 2009). Microtubule-associated protein light chain 3 (LC3), an Atg8 homolog in mammalian cells, is a well-known marker of autophagy that localizes in autophagosome membranes (Kabeya et al., 2000). The conjugation of LC3 to phosphatidylethanolamine (PE) on the early autophagosome structure is a critical step of autophagy (Ichimura et al., 2000).

Selective autophagy-mediated clearance of invading microbes is becoming recognized as a potent host immune response (Huang and Brumell, 2014). *Salmonella enterica* serovar Typhimurium has been well-studied as a model intracellular bacterium subjected to autophagy (Narayanan and Edelmann, 2014). Recognition of *S*. Typhimurium by the host autophagy machinery is thought to be mediated mainly by two signals: ubiquitin, which is associated with cytosolic bacteria or bacteria-containing vacuoles (Birmingham et al., 2006), and a sugar β-galactoside on the luminal surface of bacteria-containing vacuoles that can be exposed by membrane rupture and is sensed by cytosolic receptor galectin-8 (Thurston et al., 2012). Recruitment of autophagic machinery to bacteria or bacterial vacuoles is mediated by LC3-binding adaptor proteins like p62/SQSTM1 and Nuclear dot protein 52 kDa (NDP52) (Thurston et al., 2009; Deretic, 2010; Cemma et al., 2011). In addition to an LC3 binding site, p62 carries a ubiquitin binding site (Zheng et al., 2009), and NDP52 has both ubiquitin and galectin-8 binding sites (Thurston et al., 2012). Another type of adaptor protein, Tecpr-1, which interacts with autophagy proteins Atg5 and WIPI2 (Atg18 homolog in mammalian cells), does not interact with ubiquitin or galectin-8 (Ogawa et al., 2011).

To counteract autophagy, bacteria have developed evasion strategies. The *S*. Typhimurium effector protein SseL deubiquitinates proteins on *Salmonella*-containing vacuoles (SCVs), preventing recognition by autophagic machinery (Mesquita et al., 2012). The *L. pneumophila* effector RavZ irreversibly deconjugates LC3 from PE (Choy et al., 2012), robustly inhibiting host autophagy. However, even in the absence of RavZ, *L. pneumophila* does not recruit LC3 on the vacuole (Hubber et al., 2017) and is highly resistant to clearance by selective autophagy, suggesting that *L. pneumophila* has alternative mechanisms for autophagy evasion (Choy et al., 2012; Rolando et al., 2016). The fact that *L. pneumophila* rarely recruits LC3 even in the absence of RavZ makes it difficult to identify alternative *L. pneumophila* factors inhibiting LC3 recruitment to LCVs. In an attempt to explore the possible alternative mechanisms, we used SCVs to examine potential roles of *L. pneumophila* effector proteins related to inhibition of selective autophagy. Using co-infection with *L. pneumophila* and *S*. Typhimurium, we unexpectedly found that RavZ plays a role in interfering with ubiquitin recruitment to the *Salmonella*-containing vacuoles.

## Materials and methods

### Bacterial strains, plasmids, and culture

*S. Typhimurium* SR-11 x3181 carrying an mCherry-expressing plasmid (Hubber et al., 2014) was used for infection throughout the study. *Legionella* strains used in this study were derivatives of *L. pneumophila* strains Philadelphia-1 (Lp01) (Berger and Isberg, 1993). *Salmonella* was grown in Luria-Bertani (LB) medium containing 20 μg/ml of chloramphenicol to maintain the mCherry expressing plasmid. *Legionella* strains were grown in liquid N-(2-acetamido)-2-aminoethanesulfonic acid (ACES) buffered yeast extract (AYE) media (Horwitz and Silverstein, 1983). The *ravZ* and *dotA* deletion strains were constructed by allelic exchange as described previously (Zuckman et al., 1999). Eukaryotic expression plasmids for RavZ were constructed by cloning the PCR-amplified wild-type or mutant (C258A) *ravZ* into the p3xFLAG-CMV-10 expression vector (Sigma). Site-directed mutagenesis was conducted using the Quickchange II Site-Directed Mutagenesis Kit according to the manufacturer's recommendations (Agilent Technologies).

### Cell cultures and transfection

HeLa-FcγRII and HEK293T-FcγRII cells were grown in Dulbecco's modified Eagle medium supplemented with 10% fetal calf serum. Bone marrow derived macrophages (BMDMs) were prepared from A/J mice (Japan SLC, Inc.). The cells were plated onto cover glass in 24-well tissue culture plates 1 day before infection or transfection. Transfection was conducted using Lipofectamine 2000 (Invitrogen) or Fugene HD (Promega) according to the manufacturers' recommendations.

### Antibodies

Mouse antibody against mono- and polyubiquitinylated conjugates (FK2) was purchased from Enzo (BML-PW8810). Mouse antibody against ubiquitin (P4D1) was purchased from Cell Signaling (#3936). Mouse antibody against α-tubulin was purchased from Sigma (T6074). Rabbit anti-*Legionella* antibody was purchased from Biodesign (B65051G). Secondary antibodies were purchased; Peroxidase-conjugated goat anti-mouse antibody (62–6520, Invitrogen), Alexa Fluor 488-conjugated goat anti-mouse antibody (A-11029, Thermo Fisher Scientific) and Rhodamine RedX-conjugated goat anti-rabbit antibody (R6394, Thermo Fisher Scientific).

### Bacterial infections

Overnight *Salmonella* culture was diluted 1:30 with fresh LB medium containing 20 μg/ml of Chloramphenicol, grown to an OD_600_ value of about 2.0, and used for infection. Overnight liquid culture of *Legionella* was used for infection. The bacterial cultures were spun down by microfuge, the culture media were replaced with phosphate-buffered saline (PBS), and the suspension was immediately used for infection. HeLa cells were infected with *Salmonella* with multiplicity of infection (MOI) 300 for 1 h. After 20 min of infection, the cell culture medium was replaced with fresh medium containing 50 μg/ml of gentamycin to kill extracellular *Salmonella*. For the co-infection, a pre-mixed solution of *Salmonella* and *Legionella* was prepared and immediately used for infection with MOI 300 of both bacteria for 1 h. The time course study of *Legionella* infection in BMDMs was conducted with MOI 50. After 1 h of infection, the cells were washed three times with PBS to remove extracellular *Legionella* and the culture medium was replaced with fresh medium.

### Immunostaining

Infected cells were fixed with 4% (w/v) of paraformaldehyde (PFA) after three washes with PBS and permeabilized with chilled methanol or with 0.1% (w/v) of Triton X-100 in PBS. After blocking with 2% (w/v) of goat serum in PBS for 20 min, the cells on the coverslips were reacted with a primary antibody for 1 h, followed by a secondary antibody for 30 min, at concentrations recommended by the manufacturers.

### Protein purification

BL21(DE3) carrying pET15b-*ravZ* (pNH1678) or pET15b-*ravZ*_C258A_ (pNH1679) was grown to logarithmic phase at 37°C in 1 l of L-broth. After addition of IPTG to a final concentration of 0.4 mM to induce the production of His-RavZ, cells were cultured overnight at 16°C. *E. coli* cells were recovered by centrifugation and suspended in buffer A (20 mM Tris HCl pH 7.5, 5 mM EDTA) containing complete protease inhibitor cocktail (Roche). After treatment with 250 μg/ml lysozyme for 30 min, cells were disrupted by sonication. Lysate was centrifuged (20,000 g, 20 min) to remove insoluble materials and applied to Q-sepharose Fast Flow (~25 ml bed volume; GE Healthcase) equilibrated with buffer B (20 mM Tris HCl pH 7.5, 10 mM β-mercaptoethanol) in an open column format. After washing with buffer B containing 200 mM NaCl, the fraction containing His-RavZ was eluted with buffer B containing 300 mM NaCl. This fraction was further applied to a HisSelect column (1 ml bed volume; Sigma-Aldridge) equilibrated with buffer C (20 mM Tris HCl pH 7.5, 300 mM NaCl, 10 mM β-mercaptoethanol). After washing with buffer C containing 10 mM imidazole, fractions were eluted with a linear gradient of 10–100 mM imidazole in buffer C. Fractions containing His-RavZ were pooled and concentrated and subjected to a size exclusion column (Superdex 200 16/60 pg; GE healthcare). His-RavZ was eluted in 20 mM Tris HCl pH 7.5, 300 mM NaCl, 1 mM DTT.

### Semi-permeabilized cells experiment

After 1 h of infection with *Salmonella*, HeLa cells were washed twice with semi-permeabilization buffer [125 mM K(OAc), 2.5 mM Mg(OAc)_2_, 25 mM Hepes-KOH pH 7.4, 1 mg/ml Glucose, 1 mM DTT]. The cells were treated with 30 μg/ml digitonin in the semi-permeabilization buffer for 4 min at room temperature and washed another three times with the buffer. The permeabilized cells were treated with purified His-tagged wild-type or mutant RavZ proteins with gentle agitation for 1 h at room temperature. After two washes, the cells were fixed with 4% (w/v) PFA and subjected to immunostaining.

### Statistical analysis

Immunofluorescence experiments were conducted with at least 100 bacterial vacuoles counted per single experiment. Values were compared using paired Student's *t*-tests on three independent experiments.

### Ethics statement

All animal experiments were performed in accordance with the institutional guidelines and were approved by the Animal Care and Use Committee of the Research Institute for Microbial Diseases, Osaka University, Japan (Biken-AP-H26-10-0).

## Results

### LC3 recruitment to *Salmonella*-containing vacuoles is inhibited by co-infecting *L. pneumophila*

**Figure 1:**
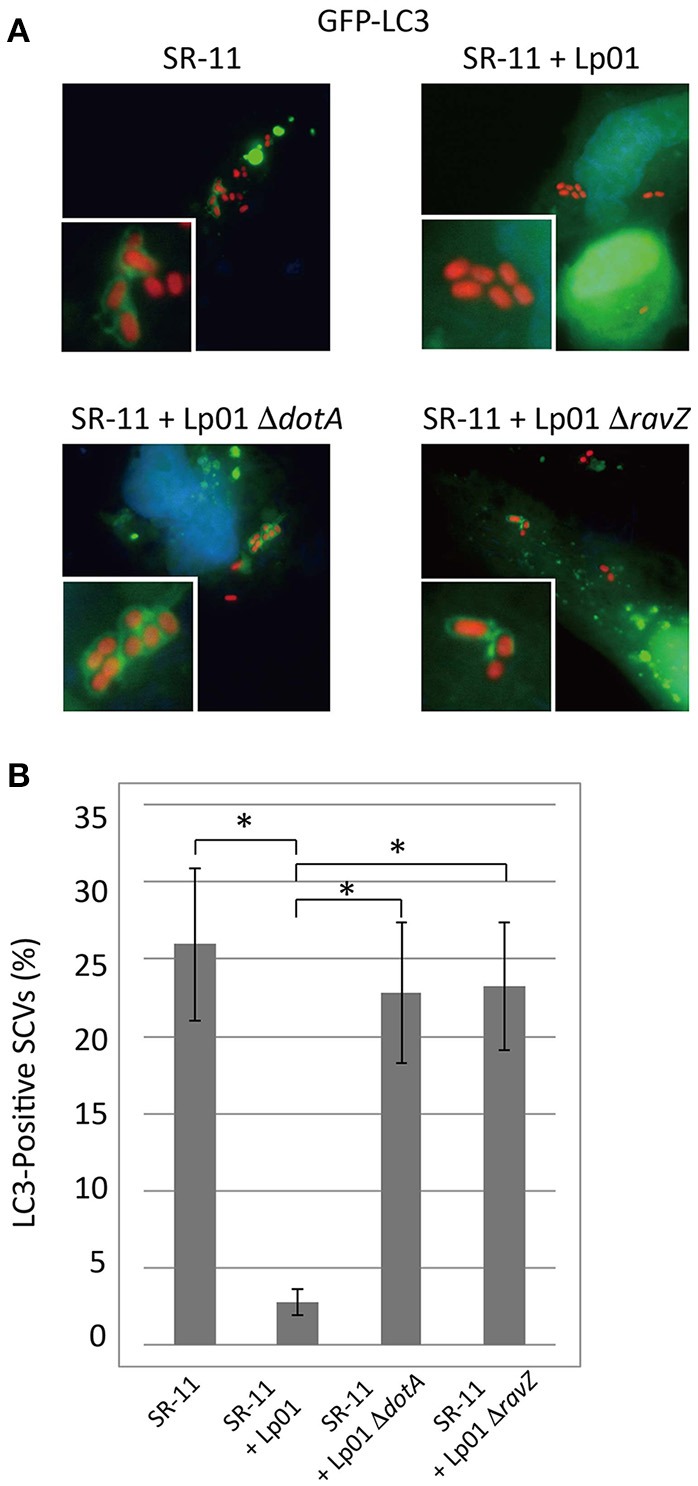
*Legionella* interferes with LC3 recruitment to SCVs in a T4SS- and RavZ-dependent manner. HeLa cells expressing GFP-LC3 were co-infected with *Salmonella* expressing mCherry and *Legionella* for 1 h. **(A)** LC3 recruitment to SCVs was visualized by fluorescence microscopy. **(B)** Average percentage of LC3-positive SCVs in the cells was calculated from three independent examinations. Data present the average ± *SD*; ^*^P < 0.02.

As previously described, *Legionella* RavZ is sufficient to inhibit macroautophagy in mammalian cells, but it is not the only determinant preventing LC3 recruitment to LCVs (Choy et al., 2012). In an attempt to identify *L. pneumophila* protein factors responsible for the prevention of LC3 recruitment, we used an *S*. Typhimurium and *L. pneumophila* co-infection system. Selective autophagy targets a fraction (~25%) of *Salmonella*-containing vacuoles (SCVs) at 1 h post-infection, as evidenced by the LC3-positive SCVs observed in GFP-LC3 expressing HeLa cells challenged with *S*. Typhimurium (Figure 1 SR-11). When HeLa cells were co-infected with wild-type *L. pneumophila*, LC3 recruitment to SCVs was reduced (Figure 1 SR-11+Lp01). In contrast, in HeLa cells co-infected with *L. pneumophila* strains lacking functional Dot/Icm T4SS or RavZ, LC3 recruitment to SCVs was not affected (Figure 1 SR-11+Lp01 Δ*dotA*/Δ*ravZ*). These results illustrate that the co-infection experiment can be used to identify a *L. pneumophila* effector responsible for the restriction of LC3 recruitment to bacteria-containing vacuoles. These results also demonstrate that the restriction of LC3 recruitment to SCVs in the experimental condition can be explained solely by the function of the *L. pneumophila* effector RavZ, and any other effectors play only a limited role.

### *Legionella* co-infection restricts p62, NDP52, and ubiquitin recruitment to SCVs

**Figure 2:**
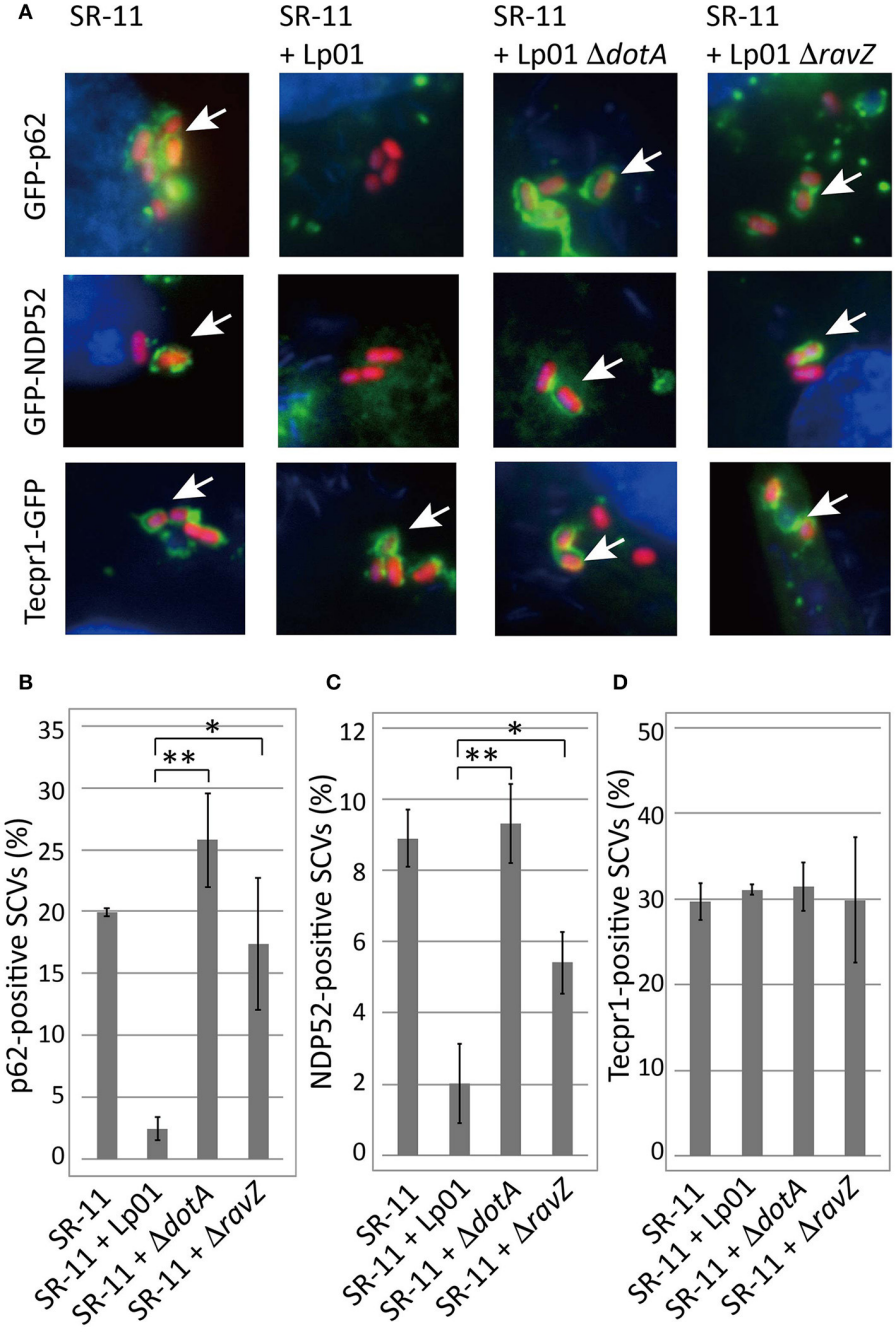
*Legionella* interferes with the recruitment of ubiquitin-dependent adaptor proteins to SCVs in a T4SS- and RavZ-dependent manner. HeLa cells expressing GFP-p62, GFP-NDP52, or Tecpr1-GFP were co-infected with *Salmonella* expressing mCherry and *Legionella* for 1 h. **(A)** Recruitment of the adaptor proteins to SCVs was visualized by fluorescence microscopy. Adaptor-positive SCVs are indicated by arrows. Average percentage of p62 **(B)**, NDP52 **(C)**, or Tecpr1 **(D)**-positive SCVs in transfected cells was calculated from three independent examinations. Data present the average ± *SD*; ^*^P < 0.02, ^**^P < 0.01.

**Figure 3:**
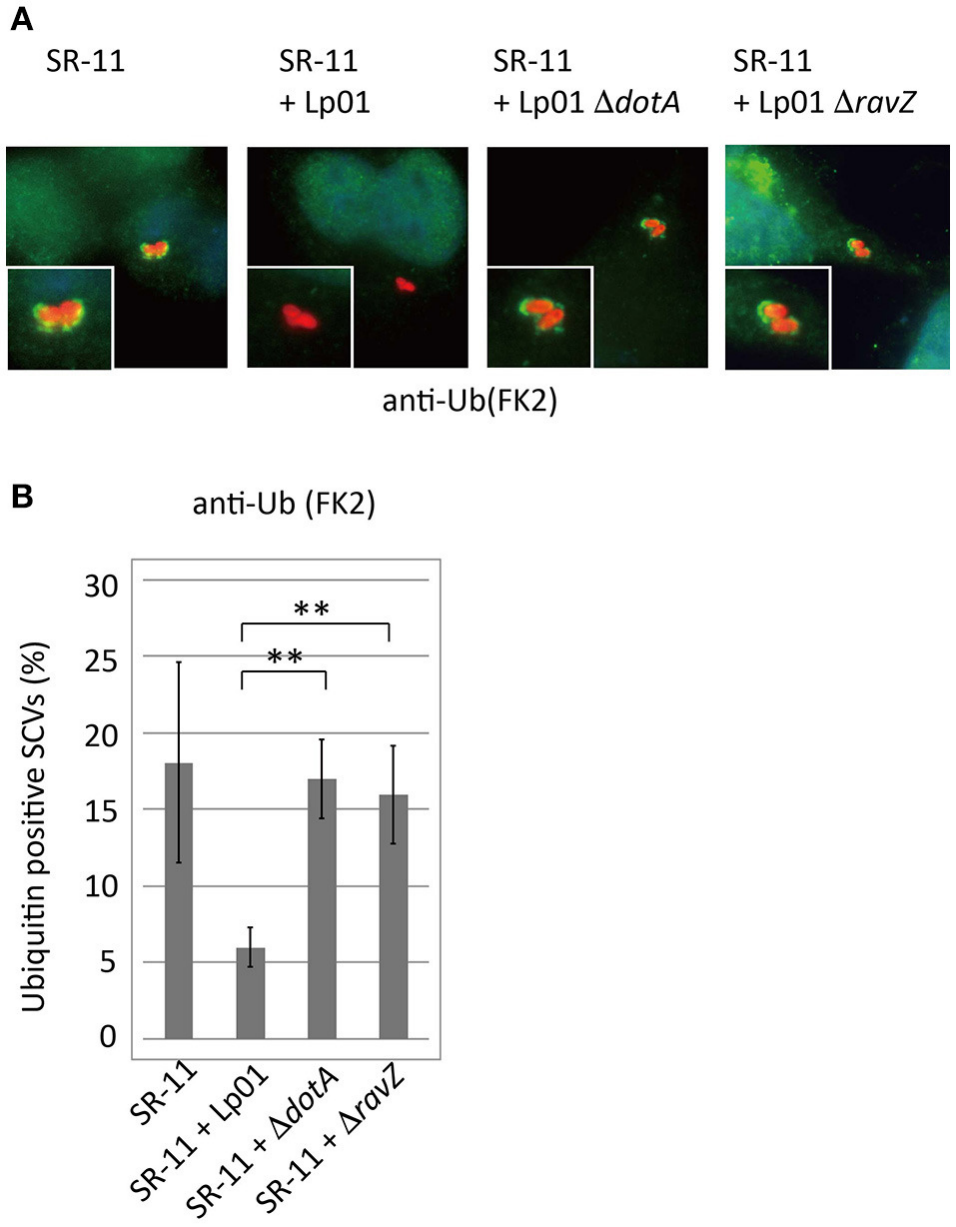
*Legionella* interferes with ubiquitin recruitment to SCVs in a T4SS- and RavZ-dependent manner. HeLa cells were co-infected with *Salmonella* expressing mCherry and *Legionella* for 1 h. **(A)** Recruitment of ubiquitin to SCVs was visualized by immunofluorescence microscopy. Conjugated ubiquitin was stained with the FK2 antibody (green). **(B)** Average percentage of ubiquitin-positive SCVs in the cells calculated from three independent examinations. Data present the average ± *SD*; ^**^P < 0.01.

Adaptor proteins such as p62 and NDP52 carry both LC3- and ubiquitin-binding domains and are proposed to play a critical role in the recognition of invading microorganisms and the induction of selective autophagy. We thus examined the effect of co-infecting *L. pneumophila* on the recruitment of these adaptor proteins to SCVs. A fraction of SCVs acquired GFP-p62 or GFP-NDP52 in HeLa cells producing GFP-p62 or GFP-NDP52 respectively at 1 h post-infection (Figure 2 SR-11). Intriguingly, the recruitment to SCVs of both GFP-p62 and GFP-NDP52 was severely restricted by co-infecting *L. pneumophila*, as in the case of LC3 (Figures 2A,B,C SR-11+Lp01). The restriction was dependent on the presence of functional Dot/Icm T4SS in the co-infecting *L. pneumophila* (Figures 2A,B,C SR-11+Lp01 Δ*dotA*). RavZ disruption restored the GFP-p62 and GFP-NDP52 recruitment to extents similar to or somewhat less than those observed in HeLa cells infected solely with *S*. Typhimurium (Figures 2A,B,C SR-11+Δ*ravZ*). We also examined the recruitment to SCVs of Tecpr1-GFP, another autophagy adaptor protein that does not possess ubiquitin-binding activity. In contrast to GFP-p62 and GFP-NDP52, Tecpr1-GFP recruitment to SCVs was not affected by co-infecting *L. pneumophila* (Figures 2A,D). These results prompted us to examine ubiquitin recruitment to SCVs, an upstream event before adaptor recruitment, in the co-infection system. Using immunofluorescence microscopy with anti-conjugated-ubiquitin antibody (FK2), we found that the fraction of ubiquitin-positive SCVs was significantly reduced in the presence of co-infecting *L. pneumophila*, (Figures 3A,B SR-11+Lp01). As in the case of LC3, p62, and NDP52, the restriction required a functional Dot/Icm T4SS and the effector RavZ of co-infecting *L. pneumophila* (Figures 3A,B SR-11+Lp01 Δ*dotA/*Δ*ravZ*).

### RavZ restricts ubiquitin recruitment to SCVs

**Figure 4:**
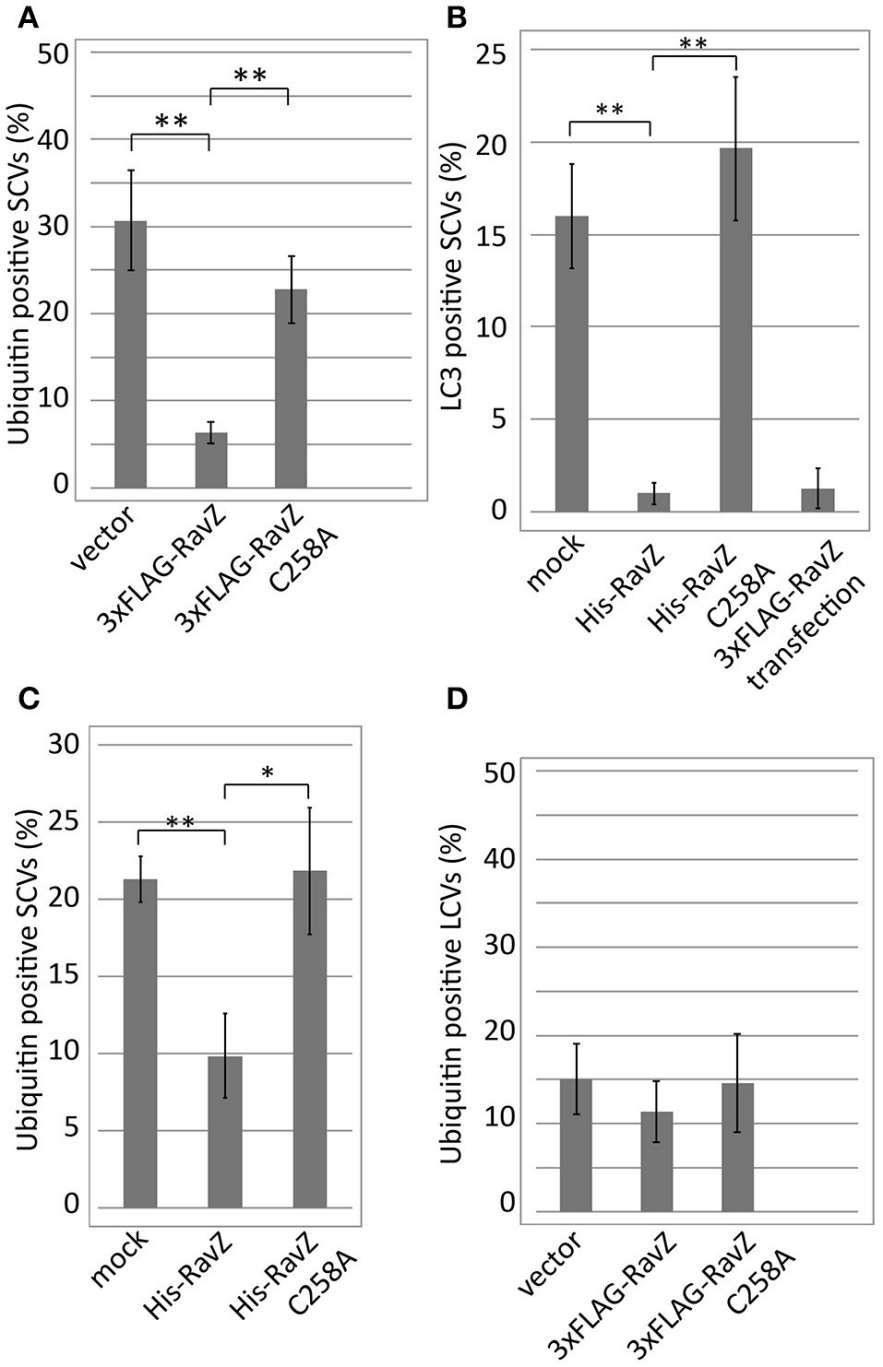
*Legionella* RavZ appears to remove ubiquitin from SCVs in a manner dependent on the catalytic residue responsible for autophagy inhibition. **(A,D)** 3xFLAG-RavZ or its catalytic mutant was expressed in HeLa cells by transfection. The cells were infected with *Salmonella* expressing mCherry **(A)** or *Legionella* **(D)** for 1 h. **(B,C)** The purified His-tagged RavZ or its catalytic mutant protein was supplied into HeLa cells expressing GFP-LC3 **(B)** or HeLa cells **(C)** semi-permeabilized with Digitonin-containing buffer after 1 h of infection with *Salmonella* expressing mCherry. Conjugated ubiquitin was stained with the FK2 antibody. Average percentage of LC3 **(B)** or ubiquitin **(A,C,D)**-positive SCVs or LCVs calculated from three independent examinations. Data present the average ± *SD*; ^*^P < 0.02, ^**^P < 0.01.

The *L. pneumophila* effector RavZ appears to be necessary for the reduction of ubiquitin-positive SCVs in the co-infection condition. To test whether RavZ was sufficient for this reduction, we examined ubiquitin recruitment to SCVs in HeLa cells ectopically expressing RavZ (Figure 4A). The fraction of ubiquitin-positive SCVs in 3xFLAG-RavZ producing HeLa cells was lower (~5%) than that in vector-transfected cells (~30%), indicating that RavZ is sufficient for the restriction. RavZ deconjugates LC3, and the RavZ_*C*258A_ mutation disrupts this enzymatic activity. Ectopic expression of 3xFLAG-RavZ_*C*258A_ did not affect ubiquitin recruitment to SCVs, demonstrating that the same enzymatic activity is required for the restriction of ubiquitin recruitment to SCVs (Figure 4A).

To further characterize the role of RavZ in decreasing ubiquitin-positive SCVs, we used semi-permeabilized *Salmonella*-infected HeLa cells. The semi-permeabilized cells were washed to remove cytoplasmic proteins and compounds, and purified His-RavZ or His-RavZ_*C*258A_ was added back at various concentrations. LC3 and ubiquitin levels on SCVs were determined by immunofluorescence staining using specific antibodies. Treatment with His-RavZ at a saturating concentration of 25 μg/ml (430 μM) decreased LC3 detectable on SCVs (Figure 4B, Figure S1A). In the same condition, the fraction of ubiquitin-positive SCVs was reduced two-fold (Figure 4C). The added His-RavZ significantly reduced ubiquitin and LC3 levels on SCVs at a concentration as low as 0.008 μg/ml (140 nM), and the reactions were readily saturated as His-RavZ concentration increased (Figure S1), indicating the RavZ activity toward ubiquitin had an effective concentration comparable to that of its activity toward LC3. There was a residual fraction of ubiquitin-positive SCVs even at the saturated concentration of RavZ, suggesting that a certain fraction or species of ubiquitin cannot be removed by the action of RavZ (Figure S1B). Collectively, these results demonstrated that the activity of RavZ is necessary and sufficient for the reduction of ubiquitin levels on SCVs, presumably by enzymatically removing ubiquitin from unknown substrates on *Salmonella* or SCVs.

### RavZ does not affect ubiquitin recruitment to LCVs

**Figure 5:**
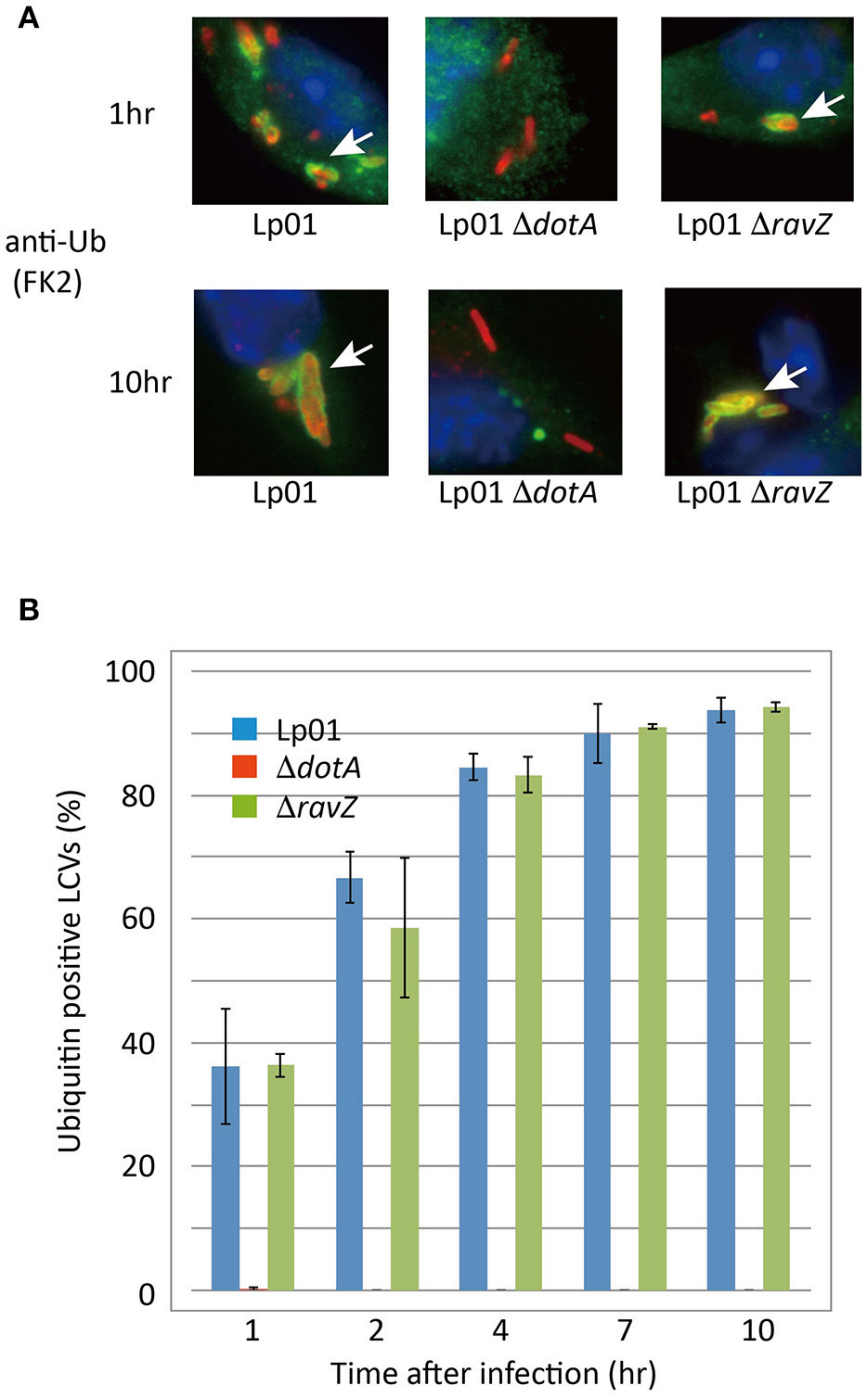
RavZ appears not to affect ubiquitin recruitment to LCVs. Mouse bone marrow-derived macrophages were infected with *Legionella* for the indicated hours. **(A)** Recruitment of ubiquitin to LCVs was visualized by immunofluorescence microscopy. *Legionella* was stained with anti-*Legionella* antibody (red). Conjugated ubiquitin was stained with the FK2 antibody (green). Ubiquitin-positive LCVs are indicated by arrows. **(B)** Percentage of ubiquitin-positive LCVs. Data present the average ± *SD* calculated from three independent experiments.

We examined whether RavZ inhibits ubiquitin recruitment to LCVs in *L. pneumophila*-infected cells. In HeLa cells infected with a *L. pneumophila* Δ*ravZ* strain, the frequency of ubiquitin-positive LCVs was not significantly affected by ectopically expressed 3xFLAG-RavZ or its catalytic mutant (Figure 4D). In mouse bone marrow-derived macrophages (BMDMs), infection with *L. pneumophila* resulted in the appearance of LCVs highly decorated with ubiquitin in a T4SS-dependent manner (Figure 5). However, up to 10 h post-infection, there was no significant difference in the fractions of ubiquitin-positive LCVs containing wild-type and Δ*ravZ* strains. It has been shown that ubiquitin recruitment to LCVs requires bacterial protein synthesis (Dorer et al., 2006). To examine the possibility that the persistent ubiquitin recruitment to LCVs obscures RavZ-dependent ubiquitin reduction, we repeated this experiment in the presence of chloramphenicol, a bacterial translation inhibitor, in order to restrict de novo supply of ubiquitin to LCVs (Figure S2). The fraction of ubiquitin-positive LCVs was strongly reduced in the presence of chloramphenicol, while again there was no significant difference in the fractions of ubiquitin-positive LCVs containing wild-type and Δ*ravZ* strains. These results do not support a potential contribution of RavZ to removing ubiquitin from LCVs.

### The RavZ-dependent reduction of ubiquitin-positive SCVs does not require a functional LC3 conjugation system

**Figure 6:**
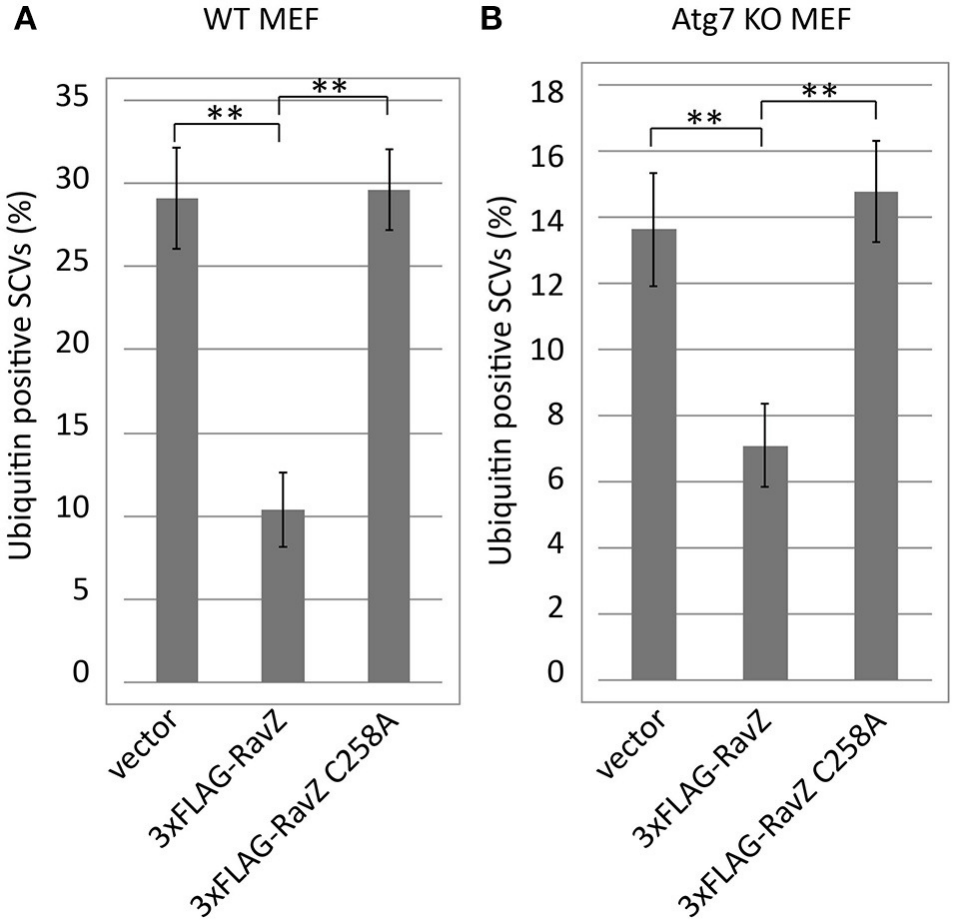
Deficiency for LC3 maturation is not a cause of RavZ-mediated ubiquitin reduction on SCVs. Wild-type **(A)** or Atg7 knockout **(B)** MEF cells expressing RavZ or its catalytic mutant were infected with *Salmonella* expressing mCherry for 1 h. Conjugated ubiquitin was stained with the FK2 antibody. Percentage of ubiquitin-positive SCVs calculated in three independent experiments. Data present the average ± *SD*; ^**^P < 0.01.

Autophagy adaptor proteins p62 and NDP52 can bind both to ubiquitin and to LC3. RavZ possesses the enzymatic activity to irreversibly deconjugate LC3 from PE. Therefore, one of the possible explanations for the reduction of ubiquitin-positive SCVs in the presence of functional RavZ is that RavZ-dependent deconjugation of LC3 leads to destabilization or dissociation of ubiquitinated complexes containing adaptor proteins in the vicinity of SCVs. Alternatively, the apparent decrease in ubiquitin could be attributed to loss of ubiquitinated LC3 from SCVs. These feedback scenarios predict that the reduction of LC3-positive SCVs is a prerequisite for the reduction of ubiquitin-positive SCVs. To test this possibility, we examined the ubiquitin levels on SCVs in Atg7 knockout (KO) MEF cells, in which LC3 conjugation to PE does not take place (Komatsu et al., 2005) (Figure 6). We found that the RavZ-dependent reduction of ubiquitin-positive SCVs was observed both in wild-type and Atg7 KO MEF cells (Figure 6). This strongly suggests that the RavZ-dependent restriction of ubiquitin-positive SCVs does not result from the RavZ-mediated deconjugation of LC3 from PE.

## Discussion

Autophagy is carried out by a set of Atg proteins. Among the ~30 Atg proteins, the ubiquitin-like proteins Atg12 and Atg8 have important roles in the process (Geng and Klionsky, 2008). Similarly to the proteins involved in ubiquitin conjugation reactions, E1 and E2-like Atg proteins (Atg7 and Atg3/10 respectively) have been identified (Shintani et al., 1999; Tanida et al., 1999; Ichimura et al., 2000; Geng and Klionsky, 2008). Atg8 is a yeast counterpart of LC3, and structural studies of Atg8 family proteins including LC3 (Kouno et al., 2005) revealed structural similarity to ubiquitin (Shpilka et al., 2011). Atg8-PE conjugation is a crucial step for autophagosome maturation and is a reversible by the deconjugation enzyme Atg4 (Nakatogawa et al., 2007). RavZ was found to possess Atg4-like activity to deconjugate Atg8 family proteins from PE. There were several critical differences in their enzymatic activities. RavZ cleaves a distinct amide bond of LC3-PE from that targeted by Atg4 (Choy et al., 2012). Therefore, LC3 deconjugation by RavZ is an irreversible process in which deconjugated LC3 is no longer available for reconjugation to PE. Furthermore, RavZ cleaves LC3 conjugated to PE but not to another protein such as YFP, whereas Atg4 can cleave both LC3-PE and LC3-YFP.

The experiment using semi-permeabilized cells demonstrated that pre-existing ubiquitin on SCVs can be targeted by RavZ and that the reduction in ubiquitin-positive SCVs is dependent on the active site of RavZ, which is known to be responsible for LC3 delipidation (Figure 4). Furthermore, the effect was observed in the absence of LC3 conjugation to SCVs (Figure 6). One possible explanation for these results is that the known amide-bond-cleavage activity of RavZ targets not only LC3-PE but also ubiquitin-conjugated molecules on SCVs. The recently solved crystal structure of RavZ suggests mechanisms for how RavZ preferentially targets the early autophagosomal membrane to cleave lipid-conjugated LC3 (Horenkamp et al., 2015). The structure contained a catalytic domain that showed structural similarity with Ulp family deubiquitinase-like enzymes and a C-terminal domain including a phosphatidylinositol 3-phosphate (PI3P) binding site. These and our results raise the possibility that RavZ has deubiquitinase-like enzymatic activity depending on binding to PI3P present on bacterial vacuoles. A recent report about chemical approaches using semisynthetic LC3 proteins with various modifications described that RavZ activity is strictly dependent on the lipid structure of the substrate (Yang et al., 2017). Together with the fact that RavZ recognizes the LC3 conjugated to a phosphoglycerolipid PE but not LC3-protein conjugates (Choy et al., 2012), it is tempting to speculate that RavZ may have activity toward ubiquitin conjugated to some phosphoglycerolipids containing an amine group, for example PE and phosphatidylserine (PS) in bacteria-containing vacuoles. Cellular polyubiquitin levels are not affected by ectopic expression of RavZ, indicating that RavZ is not likely to be a canonical deubiquitinase (Figure S3). Such a ubiquitin-lipid conjugate might play a role as a signal for selective autophagy. Although this is an intriguing hypothesis, the possibility still remains that RavZ might play an indirect role in ubiquitin removal from SCVs, in a manner dependent on the same active site for LC3 deconjugation but not on the removal of LC3. It was reported that a subset of cytosolic *Salmonella* associates with autophagy proteins (Yu et al., 2014). In this context, ubiquitin or LC3 located on not only bacterial vacuoles but also cytosolic bacteria might be targeted by RavZ activity. Future studies to clarify the molecular mechanisms of ubiquitin removal from bacterial-containing vacuoles mediated by RavZ may shed light on the recognition process of selective autophagy, an important host response for restricting invading microorganisms.

## Author contributions

TK and HN designed research; TK, XB, AH, and HN performed research; TK, XB, and HN analyzed data; and TK and HN wrote the paper.

### Conflict of interest statement

The authors declare that the research was conducted in the absence of any commercial or financial relationships that could be construed as a potential conflict of interest.

## Supplementary material

Figure S1 Concentration-dependency of the effect of RavZ treatment of semi-permeabilized cells on the level of ubiquitin-positive SCVs. After 1 h of infection with Salmonella expressing mCherry, HeLa cells expressing GFP-LC3 were semi-permeabilized with Digitonin-containing buffer and treated with purified His-RavZ at the indicated concentrations. Conjugated ubiquitin was stained with the FK2 antibody. Percentage of LC3 **(A)** or ubiquitin **(B)**-positive SCVs calculated from three independent experiments. Data present the average ± SD.

Figure S2 RavZ appears not to affect ubiquitin recruitment to LCVs even in the absence of de novo supply of ubiquitin on LCVs. Mouse bone marrow-derived macrophages were infected with Legionella for 7 hs in the absence (dark gray) or presence of 25 μg/ml of chloramphenicol (light gray). Conjugated ubiquitin was stained with the FK2 antibody. Average percentage of ubiquitin-positive LCVs was calculated from three independent examinations. Data present the average ± SD.

Figure S3 Cellular polyubiquitin levels are not affected by RavZ. HEK293-FcγRII cells were transfected with p3xFLAG-CMV-10 expression vector or derived 3xFLAG-RavZ or RavZ C258A expressing plasmids for 24 h. Cell lysates were applied on 10–20% gradient polyacrylamide gel electrophoresis followed by immunoblotting using anti-ubiquitin antibody (P4D1) for detection of mono and polyubiquitins and anti-α-tubulin for internal loading control.
