# Supplementary figures and images for: Legionella RavZ Plays a Role in Preventing Ubiquitin Recruitment to Bacteria-Containing Vacuoles
Source: Front Cell Infect Microbiol. 2017 Aug 28;7:384. doi: 10.3389/fcimb.2017.00384 (PMC5609559; doi:10.3389/fcimb.2017.00384)

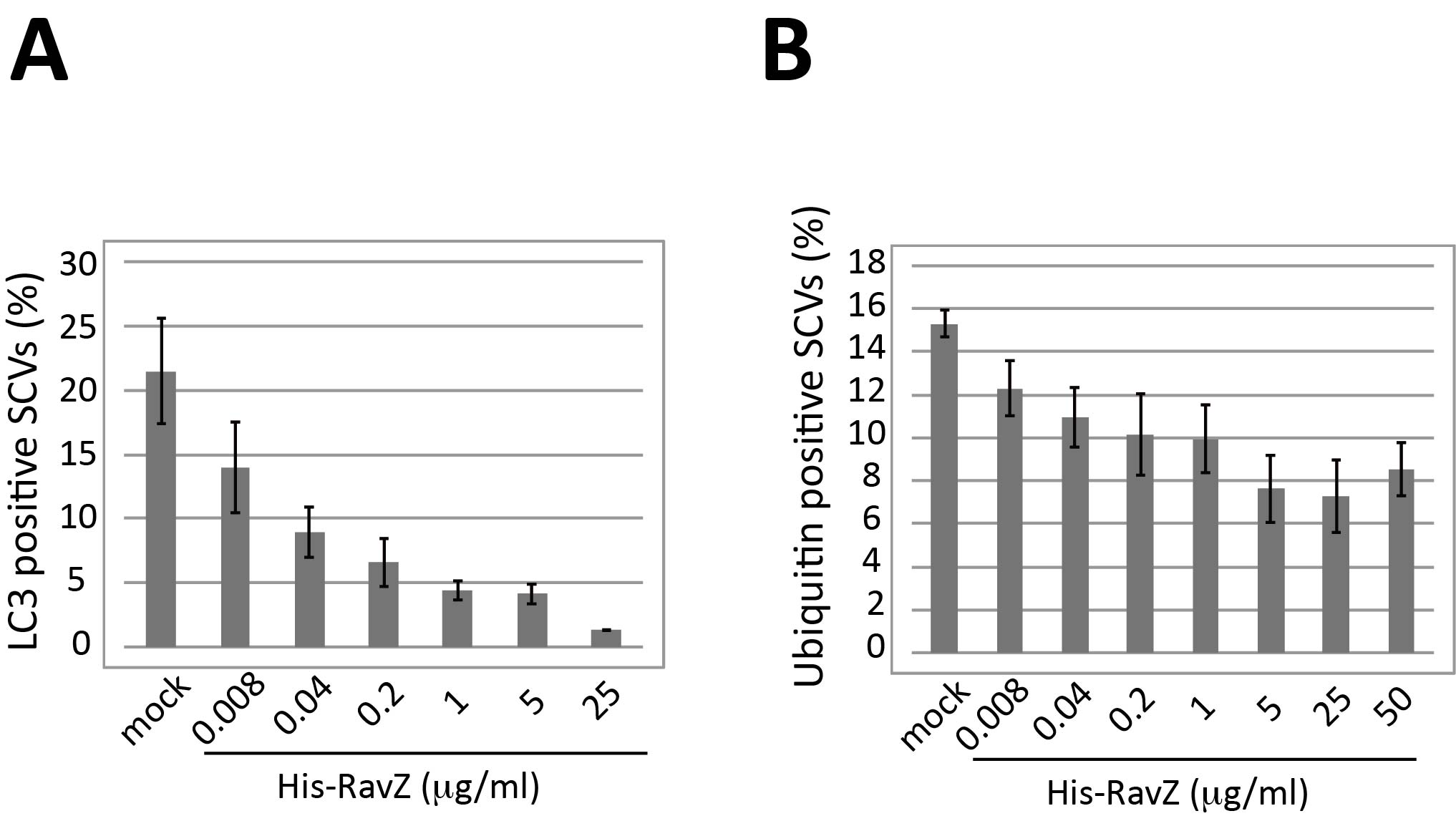

Supplement: Figure S1 — Concentration-dependency of the effect of RavZ treatment of semi-permeabilized cells on the level of ubiquitin-positive SCVs. After 1 h of infection with Salmonella expressing mCherry, HeLa cells expressing GFP-LC3 were semi-permeabilized with Digitonin-containing buffer and treated with purified His-RavZ at the indicated concentrations. Conjugated ubiquitin was stained with the FK2 antibody. Percentage of LC3 (A) or ubiquitin (B)-positive SCVs calculated from three independent experiments. Data present the average ± SD. [file Image1.JPEG]

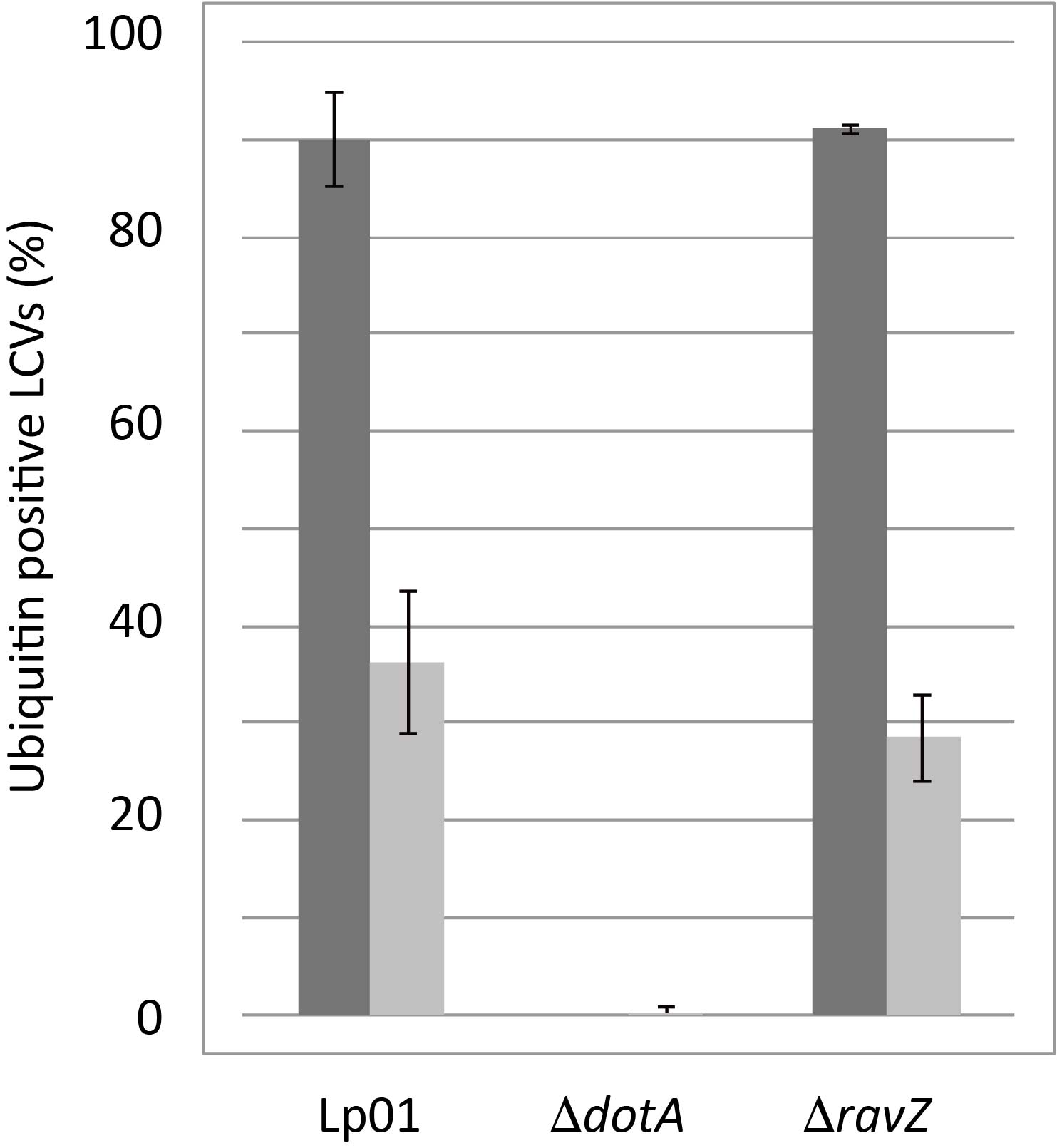

Supplement: Figure S2 — RavZ appears not to affect ubiquitin recruitment to LCVs even in the absence of de novo supply of ubiquitin on LCVs. Mouse bone marrow-derived macrophages were infected with Legionella for 7 hs in the absence (dark gray) or presence of 25 μg/ml of chloramphenicol (light gray). Conjugated ubiquitin was stained with the FK2 antibody. Average percentage of ubiquitin-positive LCVs was calculated from three independent examinations. Data present the average ± SD. [file Image2.JPEG]

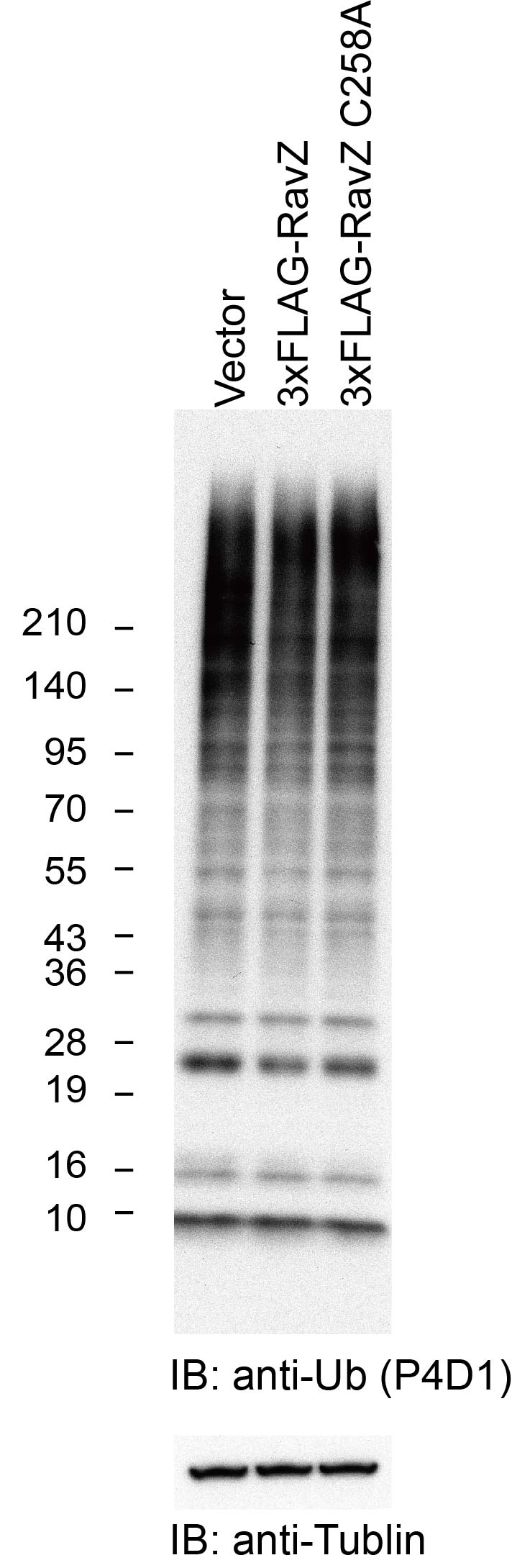

Supplement: Figure S3 — Cellular polyubiquitin levels are not affected by RavZ. HEK293-FcγRII cells were transfected with p3xFLAG-CMV-10 expression vector or derived 3xFLAG-RavZ or RavZ C258A expressing plasmids for 24 h. Cell lysates were applied on 10–20% gradient polyacrylamide gel electrophoresis followed by immunoblotting using anti-ubiquitin antibody (P4D1) for detection of mono and polyubiquitins and anti-α-tubulin for internal loading control. [file Image3.JPEG]
